# Transcultural psychiatry: Comparison of eastern and western culture and their influence on mental health and its treatment

**DOI:** 10.1192/j.eurpsy.2021.864

**Published:** 2021-08-13

**Authors:** S.P. Tiong, S. Rajkumari, N.F.B. Rasidi, O.V. Poplavskaya

**Affiliations:** Department Of Psychiatry, Narcology And Psychotherapy, Volgograd State Medical University, Volgograd, Russian Federation

**Keywords:** transcultural psychiatry, East vs. West, Cultural beliefs, Modern medicine

## Abstract

**Introduction:**

In a globalizing world, the need for humans to understand one another is fundamental. Transcultural Psychiatry aims to bridge the differences due to culture, norms and values between doctors and patients.

**Objectives:**

To study the beliefs of Eastern and Western populations on the subject of religion, the paranormal and its relation to mental health.

**Methods:**

The study was conducted targeting citizens of Eastern and Western countries (target sample size 200). A survey and 2 case studies were distributed, aimed to determine respondent’s level of belief in cultural superstitions and practices, views on mental disorders and opinions on treatment. A chi-square statistical test (significance set at ≤0.05) was performed to test validity.

**Results:**

are tabulated in Table 1. In the case studies, P-value =4.68x10^-6^ proves a strong relationship between East/West populations and their viewpoints on mental illness vs. possession. There is a strong relationship (p=3.37x10^-5^) between respondents’ beliefs in spiritual healing and its effectiveness in treating mental illness.

**Table:** 

Table 1		
	East	West
Total respondents (226)	58%	42%
Identified as religious	74%	26%
Strong belief in paranormal	85%	15%
Effectiveness of spiritual healing	55%	45%
Preferred methods of treatment		
Mental health professionals	54%	46%
Religious healer/psychic	90%	10%

**Conclusions:**

The study revealed that Eastern populations are far more superstitious and religious than their Western counterparts, and also have higher belief in the effectiveness of spiritual healing to treat mental disorders. This difference demonstrates the importance of integrating culture into diagnosis and treatment of mental illnesses, and further explore methods for more inclusive treatment plans.

